# Research progress of endogenous hematoma absorption after intracerebral hemorrhage

**DOI:** 10.3389/fneur.2023.1115726

**Published:** 2023-03-10

**Authors:** Peijie Fu, Manqing Zhang, Moxin Wu, Weixin Zhou, Xiaoping Yin, Zhiying Chen, Chuanjun Dan

**Affiliations:** ^1^Department of Neurology, Clinical Medical School of Jiujiang University, Jiujiang, Jiangxi, China; ^2^Jiujiang Clinical Precision Medicine Research Center, Jiujiang, Jiangxi, China; ^3^Medical College of Jiujiang University, Jiujiang, Jiangxi, China; ^4^Emergency Department, Affiliated Hospital of Jiujiang University, Jiujiang, Jiangxi, China

**Keywords:** phagocytosis, microglia, scavenger receptor, intracerebral hemorrhage, haematoma absorption

## Abstract

Non-traumatic intraparenchymal brain hemorrhage is referred to as intracerebral hemorrhage (ICH). Although ICH is associated with a high rate of disability and case fatality, active intervention can significantly lower the rate of severe disability. Studies have shown that the speed of hematoma clearance after ICH determines the patient's prognosis. Following ICH, depending on the hematoma volume and mass effect, either surgical- or medication-only conservative treatment is chosen. The goal of promoting endogenous hematoma absorption is more relevant because surgery is only appropriate for a small percentage of patients, and open surgery can cause additional trauma to patients. The primary method of removing hematoma after ICH in the future will involve understanding how to produce and manage macrophage/microglial endogenous phagocytic hematomas. Therefore, it is necessary to elucidate the regulatory mechanisms and key targets for clinical purposes.

## 1. Introduction

Intracerebral hemorrhage (ICH) refers to non-traumatic intraparenchymal brain hemorrhage, which is generally caused by long-term hypertension and arteriosclerosis. A sudden rise in blood pressure can result in vascular rupture and bleeding, and the blood can enter the brain parenchyma, where it solidifies and causes a mass effect. Subsequently, cells with phagocytic function engulf these blood clots. Blood clots are chemical accumulations of red blood cells (RBCs) and their lysis products such as hemoglobin (Hb), heme, iron, and globin. The pathological mechanisms of brain injury caused by these clots in the brain parenchyma include the inhibition of cell metabolism, inflammatory response, iron overload, oxidative stress, and tissue edema. Currently, hematoma-scavenging craniectomy or minimally invasive hematoma removal is the primary treatment approach for rapid hematoma clearance. However, the surgical approach has not yet been able to dramatically improve the long-term neurological prognoses in such cases. There are strict surgical guidelines, for example, hematoma-scavenging craniectomy requires craniotomy and can result in additional secondary injury. It is frequently used for patients with significant cerebral bleeding. Although minimally invasive hematoma removal can prevent craniotomy, it may result in incomplete removal of the hematoma, particularly when there is active bleeding. Accelerating the absorption of endogenous hematomas is another crucial strategy to inhibit nerve damage secondary to hematomas and for their complete removal. It has been discovered that peroxisome proliferator-activated receptor (PPAR) and nuclear factor erythroid 2-related factor 2 (Nrf2) signaling pathways can control the expression of scavenger receptor genes and activate microglia to promote endogenous hematoma absorption (1, 2). Promoting the expression of CD36 and CD163 on microglia/macrophages can enhance the phagocytosis of Hb, whereas blocking CD47 on red blood cells can promote the phagocytosis of RBCs. In addition, endogenous hematoma absorption can be facilitated by several mechanisms that increase the phagocytic capability of macrophages and microglia. Based on the current status of ICH research and treatment, this review intends to identify specific targets, explain the relevant mechanisms of endogenous hematoma absorption, and propose novel ideas for the treatment of endogenous hematoma resorption.

## 2. Cells involved in phagocytosis after ICH

In a mouse model of ICH with autologous blood injection, macrophages and dendritic cells were found to be congregated within hours of ICH (3). There is a slight increase in the percentage of dendritic cells in the hematoma compared to that in the peripheral blood of patients with ICH (4). Among the various types of phagocytic cells, macrophages/microglia remain primarily researched for phagocytosis of hematoma components following ICH (5, 6). Essentially, there are two types of macrophages. Tissue-resident macrophages are highly specialized cells that perform specific functions in the tissues where they settle, such as microglia in the central nervous system, perivascular macrophages, osteoclasts in bones, and intestinal macrophages in the gastrointestinal tract. The other type, the monocyte-derived macrophages (MDMs), can infiltrate tissues because of inflammation and chemokines. However, tissue-settled macrophages and MDMs have also been studied as a class. In 2018, Chang proposed that MDMs are essential for hematoma clearance and functional recovery after ICH (7). Subsequently, in 2021, marked differences were observed in the transcriptional states of MDMs and tissue-resident macrophages (microglia) in ICH, suggesting that the two have different functions. MDMs have a better phagocytic ability, as evidenced by the fact that they phagocytize RBCs around the hematoma in the majority of cases (8). The discovery of unique markers of tissue-resident macrophages and MDMs will aid in future research to determine their roles separately. To clarify whether endogenous hematoma absorption after ICH is required to target tissue-resident macrophages and MDMs, additional experimental data are necessary.

## 3. Scavenger receptors associated with endogenous hematoma phagocytosis

Scavenger receptors (SRs) are a class of architecturally varied proteins capable of recognizing a multitude of ligands on the cell surface, including pathogens and endogenous and modified host-derived molecules. In addition to serving as phagocytes and innate immune recognition receptors, SRs play a significant role in several physiological and pathological processes as inflammatory signal regulators. At least six distinct molecular types have been reported in the literature, among which CD36, CD163, CD91, SR-A (also known as CD204), and Lox-1 are connected to hematoma absorption after ICH.

### 3.1. CD36

CD36 belongs to the class B scavenger receptor family and is widely expressed in a variety of cells, such as microvascular endothelium cells, adipocytes, skeletal muscle cells, dendritic cells, smooth muscle cells, and hematopoietic cells. CD36 features as an SR involved in cell adhesion, antigen presentation, and identification and internalization of apoptotic cells (9). The majority of CD36 is expressed on microglia and mostly on Iba1+ cells with microglial morphology around the core area of the hematoma, as observed in a rat collagenase-induced ICH model (6). However, the expression of CD36 does not have an impact on hemoglobin levels within 24 h of ICH (6), and the volume of hematoma that is absorbed in the middle and late stages is much lower in CD36 gene-deficient rats than in normal rats, indicating that CD36 expression can influence the absorption rate of hematoma in these stages (6, 10). Mouse RBCs purified by density gradient centrifugation were diluted to 10^8^ cells/ml and added to cultured microglia at a ratio of 10:1 to establish an *in vitro* ICH model. Deletion of the CD36 gene led to a reduction in microglial phagocytosis of RBC (10), indicating that CD36 on microglia plays a role in promoting phagocytosis of RBC and hematoma absorption. However, it is still uncertain how CD36 expression in macrophages/microglia can influence erythrocyte phagocytosis. Notably, studies have shown that increasing CD36 expression can influence microglia to induce the M2 phenotype surrounding hematoma, which enhances their phagocytosis and anti-inflammatory effects (11). In addition, microglia are the only cells specifically identified in ICH for the CD36-mediated clearance of hematoma components. Therefore, we believe that upregulating CD36 after ICH can increase endogenous hematoma absorption; however, the precise mechanism and cell types involved remain to be investigated.

### 3.2. CD163

The hemoglobin SR CD163 is expressed on cells of the monocyte–macrophage lineage and participates in the uptake of hemoglobin–haptoglobin (Hb–Hp) complexes and promotes free Hb uptake (12, 13). Hb produced by erythrolysis in hematomas attaches to Hp once it is free, and the Hp–Hb complex can then be endocytosed by CD163-mediated phagocytes (14). In addition, increased Hb levels have been found to upregulate neuronal CD163 expression (15), although a significant association between neuronal CD163 and endogenous hematoma absorption was not confirmed in the current investigation. Leclerc et al. discovered that although there is a significant correlation between phagocyte CD163 and Hb clearance, hematoma volume in mice with CD163 gene deletion 3 days after ICH was 43.4±5.0% less than that in the wild-type mice. However, the mortality of ICH mice with CD163 ^−/−^ in 4–10 days was 66.7%, which was significantly higher than that of WT mice (33.3%), and the residual hematoma after 10 days was also higher than that of WT mice. Subsequent research has shown that CD163 is a scavenger receptor for Hp-Hb complexes and clears uncomplexed Hb under severe hemolytic conditions associated with Hp depletion. The authors believe that it plays a more important role in secondary brain injury after ICH (16). In previous studies, the time windows for CD163 to function were disregarded after ICH. Future studies can evaluate these time windows to efficiently connect the various hematoma absorption mechanisms. Future studies should focus on CD163, which has clinically meaningful therapeutic potential in post-ICH phagocytes.

### 3.3. Other related SRs

To absorb heme after erythrocytolysis, cells that express low-density lipoprotein receptor-related protein-1 (LRP1, also known as CD91) engulf the heme–hemopexin (heme–Hx) complex (17, 18). In addition, Bruton's tyrosine kinase–calreticulin–LRP1–Hx (BTK–CRT–LRP1–Hx) pathway regulated by the Toll-like receptor 7 (TLR7) agonist imiquimod simultaneously increases heme-Hx clearance (19). Toll-like receptor 9 (TLR9) promotes the clearance of hematoma and iron by activating macrophages/microglia after ICH, and the Nrf2/CD204/HO-1 pathway is involved in TLR9-induced macrophage/microglial phagocytosis (20). However, more studies are required to demonstrate that the CD91–heme–Hx pathway facilitates the absorption of endogenous hematomas because investigations on the aforementioned pathways have not been conducted at sufficient depth. The role of CD 204 and other SR after ICH must also be explored, as well as their potential involvement in the phagocytosis of hematoma components by macrophages/microglia.

## 4. Substances and methods for regulating phagocyte function

### 4.1. CD47

CD47 is a transmembrane protein that is a ligand for signal-regulating protein α (SIRPα) expressed in phagocytes, including macrophages and dendritic cells. When SIRP is activated, a signal transduction cascade that inhibits phagocytosis is activated (21). RBCs express the “don't eat me” signal through CD47, and this signal can interact with the macrophage inhibitory receptor SIRP to prevent phagocytosis (22). CD47 inhibiting antibodies can facilitate hematoma removal and alleviate brain damage in mice (23). Another study on ICH elderly rat model showed similar results. CD47 blocking antibody can promote hematoma clearance, reduce secondary injuries, and increase the number of macrophages/microglia in a hematoma (5). Furthermore, it has been proposed that the depletion of M2 microglia with clodronate liposomes can aggravate brain damage caused by ICH; thus, it was discovered that RBC CD47 expression inhibits microglial polarization to the M2 phenotype and phagocytic RBC, thereby inhibiting hematoma clearance, as these changes enhance brain damage after ICH (24). The inhibition of CD47 expression on RBC can improve hematoma absorption by phagocytes. Targeting the inhibition of CD47 expression on RBC is necessary because it can stop phagocytes from devouring other intraparenchymal cells that are not part of a hematoma.

### 4.2. Phosphatidylserine

Phosphatidylserine is a ubiquitous phospholipid located at the entrance of the plasma membrane. During apoptosis, phosphatidylserine is exposed to the outer surface of the plasma membrane, which is called phosphatidylserine eversion. It serves as a signal for the phagocyte to “eat me” and promotes phagocytosis (25). Macrophages phagocytose RBC, which are largely dependent on phosphatidylserine, and this has been observed in both patients with ICH and mouse ICH models. In addition, engulfing RBCs with phosphatidylserine eversion regulates the MDMs phenotype in humans and mice, as well as hematoma absorption and patient rehabilitation (7). In fact, many phosphatidylserine receptors are also categorized as SR, such as CD91, T-cell immunoglobulin and mucin receptor 4 (TIM 4), stabilin-1, and others (26). Furthermore, phosphatidylserine has been proposed to identify and attach to the extracellular domain of CD36, recognizing and phagocytizing senescent cells through phosphatidylserine-CD36 interaction (27). As a result, we believe that it will be particularly interesting to investigate the relationship between erythrocyte phosphatidylserine eversion and SR in ICH.

### 4.3. Heme oxygenase 1

Heme oxygenase 1 (HO-1) can catalyze heme to produce carbon monoxide, iron, and biliverdin. Zhang et al. investigated the function of HO-1 in 12-month-old mice (28) and found that intraperitoneal injection of the HO-1 inducer cobalt protoporphyrin IX in a collagenase-induced mouse ICH model can promote hematoma clearance, while the HO-1 inhibitor zinc protoporphyrin IX inhibits hematoma clearance in the later stages of ICH (7–28 days). In contrast, cobalt protoporphyrin IX-induced HO-1 expression can exacerbate secondary brain injury and neurological defects in the early stages of ICH (1–3 days). Consistent with the results of our research group, we believe that the neuroprotective effect of HO-1 begins early (12 h to 7 days), as observed in a rat autologous blood ICH model. In addition, HO-1 controls the Nrf2-ARE pathway in the ICH model by preventing Nrf2 from accessing the nucleus and stimulating the production of NF-κB and TNF-α, and the early neuroprotection of HO-1 is related to the nuclear translocation of Nrf2 and NF-κB (29). The ICH modeling techniques and the selection of HO-1 inducers could be responsible for the variations in HO-1 expression levels and physiological consequences in different phases in the ICH model. Notably, Nrf2-ARE transcription can enhance CD36 expression to encourage macrophage/microglial hematoma phagocytosis (2). Although there is no logical connection between this and the findings of our research team, the significance between them necessitates further consideration. Similarly, the PPAR-γ pathway can enhance the expression of microglia CD163 and HO-1 as well as promote hematoma absorption (30); therefore, targeting HO-1 to promote hematoma absorption after ICH requires a combination of other molecules and multiple signaling pathways. In addition, further investigation is required into the therapeutic window of targeted HO-1, ideally in conjunction with its clinical application.

### 4.4. Complement component C1q

Complement mediates the phagocytosis of apoptotic cells and cell debris (31). C1q is the serum complement component, which initiates the conventional complement activation pathway and is mainly involved in immunological and inflammatory responses. Some researchers have examined plasma C1q levels in patients with ICH and discovered that these levels were significantly higher than those in healthy individuals. Moreover, poor prognosis at 3 months can be independently predicted by plasma C1q, indicating that C1q may be a potential prognostic biomarker for ICH (32). Further research is needed to determine whether slow hematoma absorption or associated inflammatory responses are responsible for poor prognosis. Interestingly, there may be an interaction between C1q and CD91 (33) because CD91 functions as a heme receptor in the phagocytosis of heme, a component of hematoma after ICH (17). It cannot be denied that complement receptors play a role in the phagocytosis of RBCs by macrophages, which simultaneously release proinflammatory factors (34). In addition, these receptors are easily associated with secondary brain injuries. Further research is required to determine specific steps required to balance the complement-mediating phagocytosis and inflammation.

### 4.5. Hydrogen sulfide

Hydrogen sulfide (H_2_S) is a novel gas-signaling molecule. The primary enzyme in the brain that produces H_2_S is cystathionine β-synthase (CBS), and a decrease in CBS during ICH causes the downregulation of endogenous H_2_S synthesis (35). H_2_S has been studied for its ability to reduce inflammation after ICH (36) and provide neuroprotective benefits (35). In a recent study, H_2_S was found to be an endogenous regulator that mediates the sustained phagocytosis of microglia after ICH. Sulfide-quinone oxidoreductase (SQR) can oxidize CBS-derived endogenous H_2_S, which results in the reverse electron transport of mitochondrial complex I, leading to the production of superoxide, which conversely activates uncoupling protein 2 (UCP2) to promote microglial phagocytosis of RBC. In summary, the microglial CBS–H_2_S–complex I axis is essential for sustained phagocytosis following ICH (37). This study implies that starting from a redox approach, we can conduct research by concentrating on H_2_S to encourage hematoma absorption, offering a novel approach for future studies.

### 4.6. Remote ischemic conditioning

Remote ischemic conditioning (RIC) is a physical therapy method in which the limb is pressurized using a compression cuff. Usually, RIC is performed at 200 mmHg for four cycles of 5 min each, with a reperfusion break of 5 min (38–40). Research has shown that AMPK, which acts as a switch to control cellular metabolism, is essential for RIC to promote hematoma absorption (40). In an isolated perfused rat heart model, the number of delayed remote ischemic preconditioning stimuli was positively correlated with HO-1, and HO-1 was involved in cardioprotection (41). As mentioned earlier, HO-1 levels are closely associated with hematoma clearance (28–30). Although the pathological mechanisms of ICH and cardiovascular disease are different, the relationship between RIC and HO-1 indicates interesting possibilities for future research. In the latest clinical trial study, the safety of RIC in clinical patients with ICH was shown by comparing drug therapy alone and drug plus RIC for 7 consecutive days in patients with ICH. The hematoma-scavenging rate of the drug plus RIC was significantly higher than that of drug treatment alone. While this cannot directly explain the effect of RIC on absolute hematoma scavenging in patients with ICH, the higher scavenging rate of combination therapy is sufficient to show that RIC can promote hematoma clearance (42). Although it has been demonstrated that four cycles of RIC at 200 mmHg are clinically safe and successful in eliminating hematoma, it is still important to determine whether this is the optimal pressure.

### 4.7. Cerebral white matter fiber

**Figure 1:**
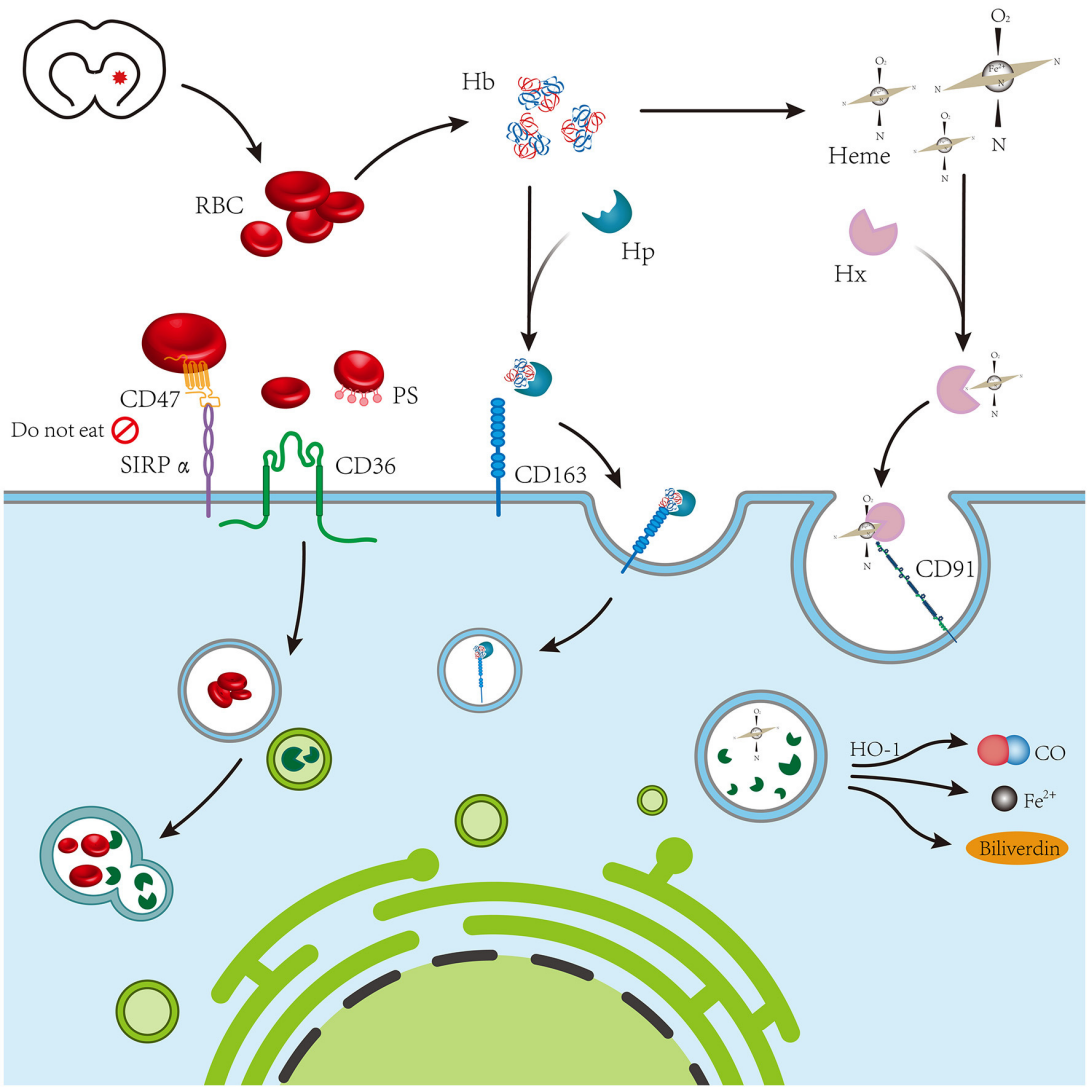
The blood chemical components of coagulation following intracerebral hemorrhage mainly include red blood cells, hemoglobin, and heme, which are mediated by CD36, CD163, and CD91 expressed on phagocytes, respectively.

Other studies have identified strategies to influence endogenous hematoma absorption after ICH. White matter fibers, for instance, are present in the core of the hematoma after ICH, and those that survive enhance the quantity of microglia/macrophages that remain there, which facilitates RBC phagocytosis and increases hematoma absorption (43). White matter fibers in the hematoma area either aid microglia/macrophages in entering the hematoma area by reducing phagocyte death or by acting as scaffolding to allow phagocytes to penetrate the hematoma area. Currently, studies on the white matter after ICH have mainly focused on white matter injury (44–47). However, the connection between white matter and endogenous hematoma absorption after ICH has not been well researched. Notably, the white matter of rodents accounts for 10–20% of brain volume, while in humans it accounts for 50% of brain volume (48), indicating that white matter plays a major role in our brain and has great research prospects in endogenous hematoma absorption after ICH. These contents are shown in Figure 1.

## 5. Signal regulation of endogenous hematoma absorption

### 5.1. PPAR-γ signaling pathway

Peroxisome proliferator-activated receptors (PPARs) are ligand-activated receptors of the nuclear hormone receptor family. There are three subtypes of ligand-induced nuclear receptors that control intracellular metabolism: PPAR-α, PPAR-β/δ, and PPAR-γ. PPAR-γ has received considerable attention in ICH research. In *in vivo* and *in vitro* ICH models, fractalkine located in neuronal cells can interact with the unique fractalkine receptor CX3CR1 and promote hematoma absorption through the PPAR-γ/CD163/HO-1 signaling pathway. Meanwhile, a study on 30 patients with ICH reported that patients with reduced hematoma had higher serum fractalkine and modified Rankin scores (mRS scores) than patients with enlarged hematoma (30). In the autologous blood injection ICH model, the PPAR-γ agonist ISO-alpha-acids (IAAs) can activate PPAR-γ and increase the expression of CD36 around the hematoma, causing microglia to polarize to the M2 phenotype, increasing endogenous hematoma absorption and decreasing inflammation around the hematoma (49). The expression of CD36 and CD163 scavenger receptors on microglia/macrophages can be simultaneously increased through the PPAR-γ signaling pathway, effectively promoting endogenous hematoma absorption. Consequently, PPAR agonists are a highly promising class of medications that merit in-depth examination.

### 5.2. Nrf2 signaling pathway

The Nrf2 signaling pathway effectively reduces leukocyte infiltration and ROS production and is essential for preventing secondary brain damage in animal models of ICH (50). In addition, Nrf2 regulates cellular phagocytosis and promotes hematoma clearance by upregulating the expression of CD36 on the surface of microglia/macrophages through ARE transcription (2). Recombinant C-C chemokine ligand17 (CCL17) promotes hematoma clearance and improves nerve injury by activating the C-C chemokine receptor 4 (CCR4)/extracellular regulated protein kinase (ERK)/Nrf2 signaling pathway to increase CD163 expression (51). Numerous mechanisms may be involved in the mutual regulation of Nrf2 and PPAR-γ gene transcription (52). In addition, research has demonstrated that the synergistic effect of PPAR-γ and the Nrf2 pathway prevents ferroptosis-induced neuronal damage in a rat ICH model (53). The effects of the interaction between PPAR-γ and Nrf2 on endogenous hematoma absorption after ICH cannot be clearly explained by the available experimental data. However, this still provides a new direction for future studies that aim to establish a perfect system for endogenous hematoma absorption after ICH by determining the relationship between various signaling pathways and their interactions.

### 5.3. Signal transducer and activator of transcription 6 signaling pathway

**Figure 2:**
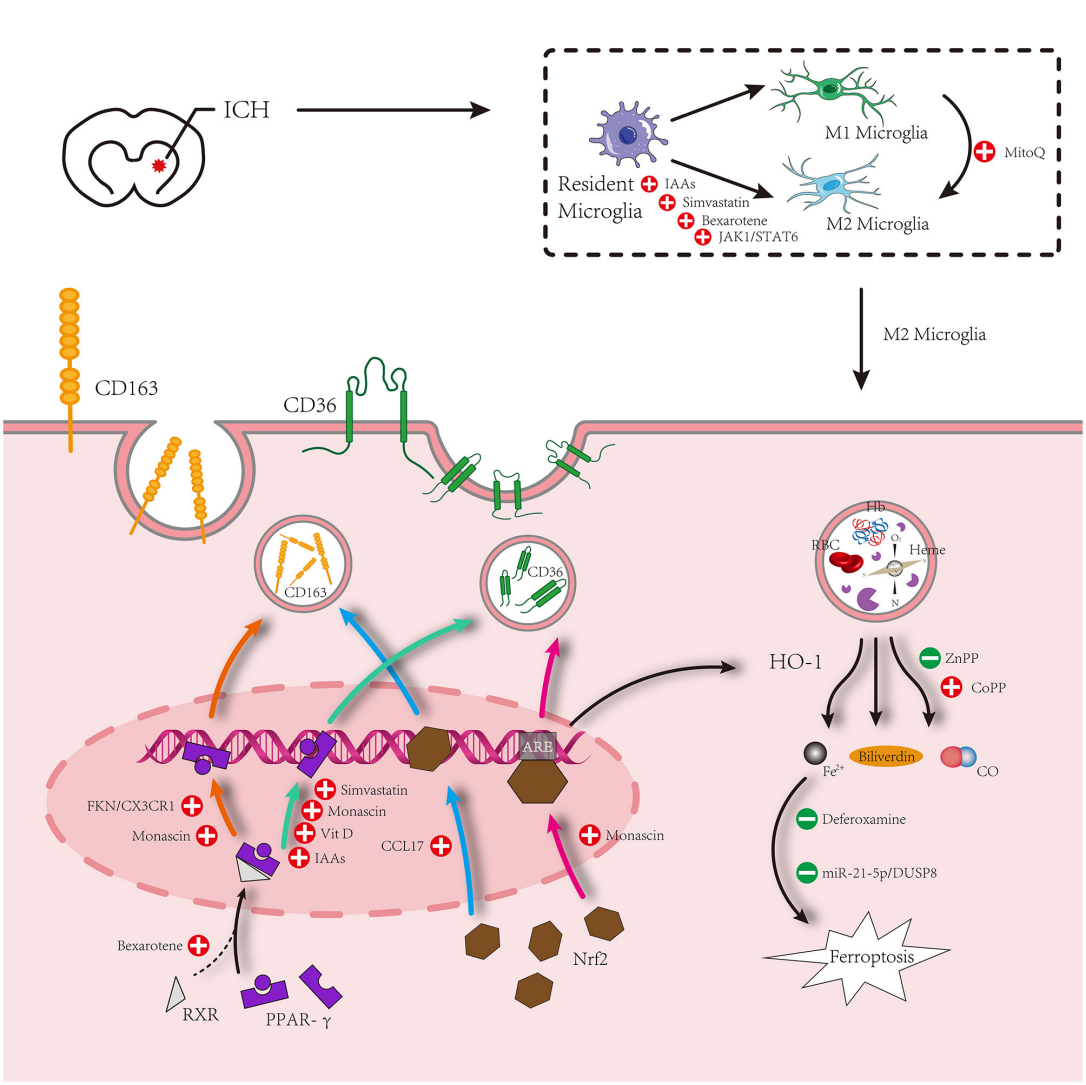
Microglia surrounding hematomas after intracerebral hemorrhage are polarized to an M1 or M2 phenotype. M1 phenotype promotes inflammation. M2 inhibits inflammation and promotes phagocytosis. The hematoma component is engulfed by M2 microglia, which mainly increases the expression of proteins related to phagocytosis under the action of PPAR-γ and Nrf2 signaling pathways and relieves the cytotoxicity of metabolites after phagocytosis of hematoma components. Simultaneously, M1 is inhibited by some other drugs to promote the polarization of M2 microglia.

The signal transducer and activator of transcription 6 (STAT6) is an important signaling pathway in macrophage function and is necessary for macrophages to evolve into M2 macrophages through an alternative pathway. Important cytokines for the polarization of macrophages to M2 include interleukin 4 (IL-4) and interleukin 13 (IL-13), which function by causing STAT6 phosphorylation and stimulating the transcription of STAT6 response genes (54). A small number of studies have demonstrated that IL-4 treatment after ICH promotes hematoma absorption, relieves neuroinflammation, and enhances neural functional recovery through the STAT6 signaling pathway (55, 56). According to one study, IL-4 can induce the polarization of M2 macrophage and microglia *via* the Janus kinase 1 (JAK1)/STAT6 pathway (55). Another study revealed that the STAT6 downstream signaling molecule STAT2 mediates the IL-4-provided function of hematoma absorption after ICH, and since STAT6 and ST2 are both necessary, the IL-4/STAT6/ST2 signaling pathway plays a crucial role in hematoma absorption after ICH (56). Nonetheless, the question remains as to whether the two signaling pathways mentioned above, IL-4/JAK1/STAT6 and IL-4/STAT6/ST2, are identical or whether they interact. However, we do not believe these two pathways are independent. In terms of effects, they both support that IL-4 promotes hematoma resolution by targeting microglia. Of course, this is only a hypothesis and needs to be verified by further experiments. The limited available data indicates that IL-4, as an anti-inflammatory factor, may induce the differentiation of anti-inflammatory microglia (also known as M2 microglia) through the STAT6 signaling pathway and mediate the phagocytosis of hematoma components. There is another signaling pathway that targets microglia-mediated hematoma phagocytosis after PPAR-γ and Nrf2, which will provide a novel idea for evaluating endogenous hematoma absorption after ICH. These contents are shown in Figure 2.

### 5.4. Regulation of microRNAs

MicroRNAs (miRNAs) are a class of small non-coding RNAs that usually target the inhibition of mRNAs to regulate the transcription and protein expression of associated genes. Through the collection of clinical data, some researchers discovered that miR-21-5p was downregulated in peripheral blood and hematoma samples of patients with ICH, with miRNA-21-5p in hematoma samples being more obviously downregulated (57), although the cause remained unknown. Using bioinformatics techniques, we found that dual-specificity phosphatase 8 (DUSP8) is a direct target of miR-21-5p. A study on the collagenase-induced rat ICH model showed inhibition of DUSP8-induced miR-21-5p activation, and involvement of the phospho-extracellular regulated protein kinase (p-ERK)/HO-1 pathway secondary brain injury. In addition, injection of a miR-21-5p antagonist can significantly inhibit ferrugination in tissues and promote hematoma absorption (58). Although some researchers have investigated how miRNAs regulate the polarization of M1 and M2 microglia after ICH (59, 60), there are no studies linking this to endogenous hematoma absorption. With the gradual development of miRNA research and the maturity of related technologies in recent years, the regulation of miRNAs to promote endogenous hematoma absorption requires more attention. Related research has accelerated new miRNA-based therapeutic strategies to promote endogenous hematoma absorption following ICH.

## 6. Drugs for promoting hematoma absorption after intracerebral hemorrhage

### 6.1. Simvastatin

Statins are a class of lipid-lowering medications. Based on recent research involving both *in vivo* and *in vitro* phagocytic models, simvastatin can upregulate CD36 and increase the polarization of M2 microglia through the PPAR-γ pathway, thereby promoting hematoma absorption after ICH (11). Simvastatin shows neuroprotective effects in ICH, and simvastatin–ezetimibe combination therapy after ICH can repair impaired nerve function and reduce inflammation (61). Simvastatin reduces the infiltration of post-ICH neutrophils into the brain parenchyma by regulating peripheral blood neutrophil apoptosis to relieve neuroinflammation (62). These may be potentially linked to endogenous hematoma absorption after ICH. The impact of simvastatin on endogenous hematoma absorption has not been studied in any relevant clinical trial, and some pertinent animal studies are required to confirm that simvastatin has the potential to be used clinically to increase endogenous hematoma absorption.

### 6.2. Deferoxamine (DFX)

Following ICH, RBCs are lysed to release Hb, which decomposes into heme under a series of pathological actions, and heme is further decomposed into iron. Deferoxamine (DFX), an iron chelator, can effectively bind iron and reduce neuroinflammation caused by iron overload (63). According to Hu et al., DFX treatment after ICH enhances heme clearance and reduces heme levels in and around hematomas through the heme–Hx–CD91 pathway (18), which is likely to be a critical step in endogenous hematoma absorption after ICH. However, studies have also demonstrated that DFX reduces erythrolysis and iron overload by inhibiting membrane–attack complexes. In addition, DFX reduces the loss of CD47 in RBC and the invasion of microglia/macrophages after ICH, which weakens RBC phagocytosis (64). Furthermore, Liu et al. revealed that DFX exerts neuroprotective effects by reducing erythrolysis and chelating iron, and inhibits Hb-induced neuronal CD163 upregulation, which may be related to the inhibition of neuronal death (15). Early clinical trials have shown that DFX mesylate therapy after ICH can inhibit encephaloedema but delay hematoma absorption (65); therefore, we are more inclined to believe that DFX has neuroprotective effects while the promoting effect on hematoma component absorption remains debatable. With the clarification of the ferroptosis mechanism and in-depth study of DFX in neuroprotection, we believe that the link between the neuroprotective effect of DFX after brain injury and the inhibition of endogenous hematoma absorption is worth exploring and has important clinical value. Nonetheless, further animal experiments are needed to provide a basis for clinical trials to verify whether DFX can be used as a drug for clinical endogenous hematoma absorption regulation.

### 6.3. Monascin

Monascin is a yellow natural pigment formed by the cultivation and fermentation of *Monascus ruber* in cereals under certain conditions, which can reduce blood lipid and blood pressure and has anti-inflammatory and antioxidant effects (66). Monascin has been shown to play a role in endogenous hematoma absorption as a natural dual agonist of Nrf2 and PPAR-γ. A high dose of monascin can promote the reduction of hematoma volume 1–7 days after ICH (67). Long-term investigations showed that Nrf2 and PPAR-γ are crucial for increasing hematoma absorption, which significantly reduces iron overload and brain atrophy after ICH (68). It has also been pointed out that the PPAR-γ agonist monascin increases the levels of haptoglobin (Hp) and CD163 on the surface of phagocytes in plasma, accelerating hematoma absorption through the Hp–Hb–CD163 pathway (1). These animal experiments have found that monascin can promote endogenous hematoma absorption to improve ICH prognosis. However, corresponding clinical data have not been collected to demonstrate the safety of monascin in the treatment of ICH. Phase I clinical studies should be conducted as the next step to verify the security and effectiveness of monascin treatment.

### 6.4. Other potential drugs

Other potential drugs that promote endogenous hematoma absorption after ICH have also been extensively studied, including wogonin (69), vitamin D (70), and ISO-α-acids (IAAs) (49), which can upregulate surface CD36 of phagocytes *via* the PPAR-γ pathway, promote the polarization of M2 type microglia, and enhance the phagocytosis of M/MΦ to promote endogenous hematoma absorption after ICH. Bexarotene has the ability to pharmacologically activate retinoid X receptor-α (RXRα) and induce nuclear translocation of RXRα and PPAR-γ, which controls the M2 phenotype of microglia, reduces neuroinflammation, and increases hematoma absorption (71). According to a recent study by Chen et al., the mitochondrial reactive oxygen species (ROS)/NLRP-3 pathway, which is also strongly linked to hematoma absorption, may promote the transition of microglia from the M1 to the M2 phenotype under the influence of MitoQ treatment (72). However, the clinical applications of these potential drugs require further research.

## 7. Prospect

Over the past years, there have been several novel research directions on endogenous hematoma absorption after ICH, such as H_2_S-mediated sustained microglial phagocytosis of hematoma, regulation of downstream signaling by miRNAs, the role of cerebral white matter fibers, and even physical therapeutic treatment methods such as remote ischemic conditioning. In conclusion, the core of hematoma absorption after ICH is the ability of phagocytes to engulf RBC. Most existing research is aimed at promoting hematoma absorption by enhancing the M2 shift of microglia and increasing the expression of their surface SRs. However, compared with other factors, PPAR-γ and Nrf2 signaling pathways that regulate leukocyte differentiation antigens on the surface of microglia to promote hematoma absorption have attracted more attention and in-depth research. Some agonists of the PPAR-γ and Nrf2 pathways can be used as clinical drugs for patients with ICH. Therefore, we believe that the regulation of microglia/macrophages will be the “final answer” to endogenous hematoma absorption after ICH. Identifying regulatory mechanisms and significant targets will be a direction for future research.

## Author contributions

ZC and CD contributed to the conception and design of the study. ZC and XY provided administrative support. PF and MW provided the study materials. PF and MW collected and assembled the data. PF, MW, and WZ performed data analysis and interpretation. MZ and ZC revised the final version. All authors contributed to the writing of the manuscript and approved the submitted version.
